# Aspects of cultivar variation in physiological traits related to Cd distribution in rice plants with a short-term stress

**DOI:** 10.1186/s40529-020-00304-3

**Published:** 2020-10-12

**Authors:** Wan-Ting Chiao, Bo-Ching Chen, Chien-Hui Syu, Kai-Wei Juang

**Affiliations:** 1 Athena Institute of Holistic Wellness, Wuyi University, Wuyishan, Fujian China; 2 Department of Natural Biotechnology, Nanhua University, Chiayi County, Taiwan, ROC; 3 Agricultural Chemistry Division, Taiwan Agricultural Research Institute, Wufeng Dist., Taichung City, Taiwan, ROC; 4 Department of Agronomy, National Chiayi University, Chiayi City, Taiwan, ROC

**Keywords:** Cadmium, Pollution-safe cultivar, Phytotoxicity, Abiotic stress, Antioxidant

## Abstract

**Background:**

Genotypic variations are seen in cadmium (Cd) tolerance and accumulation in rice plants. Cultivars that show low Cd translocation from the root into shoot can be selected to reduce Cd contamination in rice grains. This study aims to clarify the physiological regulation related to Cd absorption by rice plants for screening out the cultivars, which have relatively low Cd accumulation in grains. Eight Taiwan mega cultivars of paddy rice: japonica (TY3, TK9, TNG71, and KH145 cultivars), indica (TCS10 and TCS17 cultivars), and glutinous (TKW1 and TKW3 cultivars), which are qualified with the criteria for rice grain quality by the Council of Agriculture, Taiwan, were used for illustration. An experiment in hydroponics was conducted for the rice seedlings with a treatment of 50 μM CdCl_2_ for 7 days.

**Results and discussion:**

After the Cd treatment, the reductions in shoot growth were more significant than those in root growth; however, Cd absorbed in the rice plant was sequestered much more in the root. The malondialdehyde (MDA) was preferentially accumulated in rice root but the hydrogen peroxide (H_2_O_2_) was increased more significantly in the shoot; the antioxidative enzymes, superoxide dismutase (SOD) and ascorbate peroxidase (APX), were pronounced more in rice shoot.

**Conclusions:**

The rice cultivars preferentially accumulated Cd in the root rather than the shoot with the Cd treatment, which resulted in significant enhancements of MDA and growth reductions in the root. However, H_2_O_2_ accumulation was toward the shoot to retard shoot growth suddenly and then the root could keep a gradual growth. Also, the rice cultivars, which preferentially accumulate Cd in the root, would have the regulation tendency of SOD toward the shoot. Due to that SOD is responsible for H_2_O_2_ production, H_2_O_2_ accumulation would be thus toward the shoot. Moreover, the cultivars, which have a less regulation tendency of APX toward the shoot, would present higher translocation of Cd into the shoot.

## Background

Paddy rice (*Oryza sativa* L.) is a staple crop for most of the countries in East and Southeast Asia. The release of industrial effluents into irrigation channels that are used for flooding paddy fields and the use of phosphorus fertilizers (Cattani et al. 2008; Sebastian and Prasad 2014; Seshadri et al. 2016; Gosetti et al. 2019) result in cadmium (Cd) addition in paddy soils. The safety risk of consumption of rice due to Cd increasingly accumulated in paddy soil has been posed (Bolan et al. 2013; Zhu et al. 2016). The previous studies (Liu et al. 2007; Song et al. 2015; Duan et al. 2017) showed the large variations in Cd concentrations in plants among the different rice cultivars. Yu et al. (2006) ever proposed the concept of pollution-safe cultivars, that is, cultivars whose grains accumulate Cd levels that are low enough for safe consumption even when grown in contaminated soil. It will be a promising way for the protection of rice consumption safety to develop rice genotypes of low-Cd-accumulation in grain by breeding (Ishikawa et al. 2005; Grant et al. 2008). According to recently, understandings of Cd transport mechanisms in rice plant (Uraguchi and Fujiwara 2012; Li et al. 2017), the fast development of molecular biology resulted in the transgenic rice (Takahashi et al. 2011) and rice mutants (Ishikawa et al. 2012) to reduce the Cd uptake by plant and accumulation in grain. It will be a long term to make efforts by the genetic engineer for the development of low-Cd accumulation rice genotypes, which can take the place of the currently consuming cultivars.

Differences in Cd absorption and accumulation by the rice plants, which are grown in Cd-contaminated soil, may indicate the potential of safety risk for consuming their grains. The Cd concentration in rice grains depends on the rice plant’s Cd uptake and Cd transport from the root to shoot and from the shoot to grain (Liu et al. 2007). Thus, information about the genotypic variations of Cd tolerance, distribution, and accumulation in rice plants is useful for delineation of the safeties of rice cultivars to reduce Cd in human diets (Yu et al. 2006). In the studies, we have tried to screen the rice cultivars for selection of the genotypes that accumulated low levels of the heavy metals, such as As, Pb, and Cd, from paddy soils (Syu et al. 2014; Lai et al. 2018; Chiao et al. 2019). However, the uptake of Cd and increasing Cd concentrations in plants will result in physiological constraints that reduce the plant vigor and adversely affect growth, development, and yield. Genotypic variations in Cd distributions in rice plants and Cd accumulation in grains thus would be related to Cd stress tolerance (Zhang et al. 2009). Choppala et al. (2014) emphasized that Cd stress tolerance can be achieved by reducing the amount of Cd entering the plant cell through extracellular precipitation, biosorption to cell wall, or increased efflux. The strategies to forbidden the entrance of Cd in protoplasm would vary the distribution of Cd in rice plants.

Studies on the physiological mechanisms of Cd tolerance have provided some information about their relation to the Cd distribution in plants (Hsu and Kao 2003; Muñoz et al. 2008). Xu et al. (2013) showed that the potato leaf was the organ that showed the highest Cd accumulation, and the malondialdehyde (MDA) content increased and chlorophyll content decreased with an increase in Cd levels in the leaves. Singh and Shah (2014) found that more Cd accumulated in rice roots than in shoots; however, the stress levels of hydrogen peroxide (H_2_O_2_) and superoxide anion in the root were significantly lower than those in the shoot. This is because the activity of superoxide dismutase (SOD) in rice plants was induced more markedly in the root than in the shoot. However, the induction of SOD activity on Cd treatment associated with the stabilization of the system involved in H_2_O_2_ removals, such as the lack of ascorbate peroxidase (APX) responses to a stress burst (Olmos et al. 2003), resulted in H_2_O_2_ accumulation in cell cultures (Muñoz et al. 2008). The previous studies’ findings and current information about physiological responses to Cd stress are still not enough to clarify regulation mechanisms for Cd tolerance in rice plants. Thus, so far few concerns have been raised about the genotypic variations in Cd distribution and translocation in rice plants owing to physiological mechanisms for Cd tolerance (Mohamed et al. 2012; Xie et al. 2015). Wang et al. (2015) tried to identify genotypic variations in Cd content in rice grains depending on the physiological mechanisms for Cd tolerance. They suggested that rice cultivars have different Cd detoxification and accumulation mechanisms. Chiao et al. (2019) combined the genotypic variations of Cd absorption and physiological traits of rice seedlings to predict the Cd concentrations in rice grains of different cultivars. However, the mechanism of physiological Cd stress responses about Cd absorption and accumulation by rice plants has not yet been clarified (He et al. 2015). A physiological understanding of genotypic variations in plant Cd contents would also be useful for better selecting or breeding genotypes with low Cd accumulation in rice grains (Li et al. 2017). Therefore, the present study aims to investigate Cd absorption and toxicity in different rice cultivars and to clarify their relationship with the physiological response to Cd stress.

## Materials and methods

### Plant material and Cd treatment in hydroponics

Eight paddy rice cultivars were used in the present study, which were qualified by the criteria for rice grain quality by the Council of Agriculture, Taiwan (COA_TW 2003). These cultivars included Taoyuan No. 3 (TY3), Taikeng No. 9 (TK9), Tainung 71 (TNG71), Kaohsiung 145 (KH145), Taichung Sen 10 (TCS10), Taichung Sen 17 (TCS17), Taikeng Glutinous 1 (TKW1) and Taikeng Glutinous 3 (TKW3). TY3, TK9, TNG71, and KH145 are japonica, TCS10 and TCS17 cultivars are indica, and TKW1 and TKW3 cultivars are glutinous, which were bred from the parents of japonica rice. In Taiwan, japonica rice is the major type of rice consumed. Most of indica or glutinous rice are cultivated for food processing but not for staple consumption. Hsing (2016) reported that in Taiwan, the cultivation areas for japonica and indica rice are about 90 and 10%, respectively. In the rice breeding programs of Taiwan, the efforts on development of rice varieties are mainly for superior milling, cooking, and eating qualities, which dominate the market acceptability in priority, are used as the indexes for breeding evaluation (Hsieh 1991). The physicochemical properties of starch and protein are also drawn to set up the criteria for evaluation of rice grain quality (Lin 2006; Lur et al. 2009). At present, variation in Cd toxicity to rice plant and accumulation in rice grain is still not taken into account for Taiwan rice breeding.

The seeds of the selected rice cultivars were soaked in deionized water at 25 °C for a 36-h germination period in the dark. The germinated seeds were then transplanted in 10% modified Hoagland solution (0.5 mM KCl, 0.5 mM CaCl_2_, 0.1 mM KH_2_PO_4_, 0.2 mM MgSO_4_, 0.01 mM Fe-EDTA, and 1.5 mM NH_4_NO_3_) for a roughly 3-week growth period until they reached the morphological development stage, with three leaves completely emerged. Then the uniformly grown seedlings were drawn up for Cd treatment with 50 μM CdCl_2_ in hydroponics; a check (CK) was set at 0 μM CdCl_2_. The averages of root length and shoot length (*RL*_*0*_ and *SL*_*0*_) for the uniformly grown seedlings were also measured. The design of the treatment level was based on the previous study by Chiao et al. (2019). In the early study, Zhang and Duan (2008) have ever used the treatment of 50 µM Cd in hydroponics to investigate the genotypic difference in Cd accumulation. Recently, the same treatment level of 50 µM Cd was also used by Li et al. (2019) for investigation of Cd retention in the roots of rice seedlings. The treatment level of 50 µM Cd in hydroponics could result a significant reduction in root and shoot growths and increases of plant Cd concentrations for the different rice cultivars (Shi et al. 2005; Zhang and Duan 2008). Following a completely randomized design (CRD), the setup of hydroponic experiment was for testing variation in the cultivars with Cd treatment and for CK, respectively. There were three pots as replication for each cultivar. Twenty-five uniformly grown seedlings were planted in each pot. Next, seedlings were cultivated with Cd-spiked solutions or CK in a growth chamber for 7 days with a day/night cycle of 16/8 h at 23 °C and 27 °C, respectively, a relative humidity of 75%, and photosynthetically active radiation of 150 μmol m^−2^ s^−1^. Solution pH was maintained at 6.0 ± 0.1 with 2-(*N*-morpholino) ethanesulfonic acid buffer to ensure that Cd^2+^ was the main free ion species in solution Cd.

### Measurements of plant growth and Cd concentrations

After 7-day treatment, rice seedlings were harvested and thoroughly washed with deionized water, and the root length (*RL*_7_) and shoot length (*SL*_7_) for each cultivar were measured. Then, the root elongation (i.e., *RE* = *RL*_7_ − *RL*_0_) and shoot extension (i.e., *SE* = *SL*_7_ − *SL*_0_) were used for assessing each cultivar’s dose–response relationships for Cd toxicity. The relative root elongation (*RRE*) and relative shoot extension (*RSE*) were obtained from the ratios of root elongation and shoot extension given Cd treatment (i.e., *RE*_*Cd*_ and *SE*_*Cd*_) to those in CK (i.e., *RE*_*CK*_ and *SE*_*CK*_), respectively:

1 $$ RRE = \frac{{RE_{Cd} }}{{RE_{CK} }} \times 100\% $$

and

2 $$ RSE = \frac{{SE_{Cd} }}{{SE_{CK} }} \times 100\% $$

Harvested seedling samples were separated into the root and shoot tissues and then oven-dried at 65 °C for 72 h for determining the dry weight. Before oven-drying, extra root and shoot specimens were collected from the seedling samples for assaying the physiological traits.

Dried plant materials were digested with HNO_3_/HClO_4_ (v/v = 4/1) (Cornelissen et al. 2003), and Cd concentrations in the digests were determined using a flame atomic absorption spectrophotometer (Thermo Scientific iCE 3000 Series). Quality assurance was performed by determining the total Cd in standard reference material (SRM) No. 1573, obtained from the National Institute of Standards and Technology, USA, to audit the procedure for Cd determination in rice plants. A blank was processed for assessing the reagent interference and cross-contamination in the digestion procedure. Plant Cd concentrations in root and shoot were based on dry weight (dw). All Cd concentration measurements for each sample were repeated in triplicate.

### Determination of H_2_O_2_ and MDA contents in plant

Hydrogen peroxide contents in rice plants can be measured colorimetrically as described by Hsu and Kao (2007). The modified procedures were used for determining the H_2_O_2_ contents in rice plants. A 0.2 g fresh root or shoot sample was homogenized with 2 ml of phosphate buffer (50 mM sodium phosphate, pH 7.0) containing 1 mM hydroxylamine to extract H_2_O_2_. The homogenate was centrifuged at 12,000*g* for 20 min at 4 °C to collect the supernatant for measuring H_2_O_2_. Three replicates were obtained for the supernatant of each sample. The mixture containing 0.5 ml of supernatant and 0.25 ml of 0.1% titanium chloride (TiCl_4_) in 20% (v/v) H_2_SO_4_ was incubated at 25 °C for 5 min. Then, the absorbance at 410 nm was recorded using a microplate spectrophotometer (BioTek Epoch™) to calculate the H_2_O_2_ content using a calibration curve of standard absorbance for 10 to 100 mg L^−1^ of H_2_O_2_. The H_2_O_2_ concentration was based on fresh weight (fw).

The MDA contents were determined to evaluate the lipid peroxidation (Kosugi and Kikugawa 1985) of the rice plant tissues (i.e., root and shoot). A total of 0.2 g of fresh rice tissue was drawn and ground with liquid nitrogen into a powder that was then homogenized in 2 ml of 5% trichloroacetic acid (TCA) extraction buffer. The homogenate was centrifuged at 10000*g* for 5 min at 25 °C to collect the supernatant for measuring MDA. Next, the assay mixture containing 1.0 ml of supernatant and 4 ml of 0.5% thiobarbituric acid (TBA) in 20% TCA was incubated at 95 °C in a water bath for 30 min, and the reaction was arrested by quickly transferring the mixture into an ice bath (Mohamed et al. 2012). Finally, the absorbance of the supernatant at 532 nm and 600 nm by centrifugation at 3000*g* for 10 min was recorded by using a microplate spectrophotometer (BioTek Epoch™) to calculate the MDA content with an extinction coefficient of 155 mM^−1^ cm^−1^. The MDA concentration was based on fresh weight (fw), and MDA for each sample was determined in triplicate.

### Determination of antioxidative enzymes in plant

Antioxidative enzymes were extracted using a modified version of the procedure reported by Hsu and Kao (2004). In brief, 0.2 g of fresh rice root or shoot sample was ground with liquid nitrogen into a fine powder that was then added to 2 ml of phosphate buffer (50 mM sodium phosphate, pH 7.0) containing 1 mM hydroxylamine to extract the antioxidative enzymes (i.e., SOD and APX). The homogenate was centrifuged at 12000*g* for 20 min at 4 °C to collect the supernatant for measuring SOD and APX. Three replicates of SOD and APX measurements of the supernatants were obtained for each sample. The SOD and APX activities were measured with fresh weight (fw).

The SOD activity was determined by measuring the absorbance at 560 nm for inhibiting the photochemical reduction of nitroblue tetrazolium (NBT) according to Muñoz et al. (2008). The reaction mixture contained different dilutions of the enzyme extract in 50 mM phosphate buffer (pH 7.8), 130 mM methionine, 1 mM EDTA, and 0.63 mM NBT. The reaction was started with 7.5 μM of riboflavin by exposing it to white light for 10 min. The absorbance of the reaction mixtures at 560 nm was measured for different dilutions of the enzyme extract. These absorbance values indicated that the reductions of NBT were inhibited by the different dilutions. One unit of SOD activity was defined as the amount of enzyme per enzyme extract sample that caused 50% inhibition of the reduction of NBT (Ci et al. 2009). The absorbance values at 560 nm were recorded using a microplate spectrophotometer (BioTek Epoch™).

The APX activity was measured by the reduction of ascorbic acid in terms of the absorbance at 290 nm with an extinction coefficient of 2.8 mM^−1^ cm^−1^ according to Nakano and Asada (1981). A total of 0.1 ml of enzyme extract was added to 2.9 ml of the reaction mixture containing 1.0 ml of 50 mM phosphate buffer (pH 7.0), 0.4 ml of 0.75 mM EDTA, 1.0 ml of 1.5 mM ascorbic acid, and 0.5 ml of 6 mM H_2_O_2_. The reaction was started by adding the enzyme extract, following which the absorbance was recorded continuously every 10 s. Eventually, the decrease in absorbance at 290 nm for 1 min was measured to express the APX activity in rice tissue (Sidhu et al. 2016). The absorbance values at 290 nm were recorded using a UV–visible spectrophotometer (Thermo Scientific EVOLUTION 60S).

### Characterizations of Cd translocation and physiological regulation in plant

To investigate the Cd distribution in rice plants and the physiological regulation under Cd stress, the translocation factors (*TF*) of Cd concentration and physiological traits (i.e., SOD, APX, H_2_O_2_, and MDA) between the root and the shoot are used to show the configuration of Cd accumulation in plants and the state of physiological symptoms:

3 $$ TF = \frac{{ C_{shoot} }}{{C_{root} }} , $$

where *C*_*shoot*_ and C_*root*_ are the Cd concentrations or physiological traits observed in the shoot and root, respectively. For illustrating the Cd distribution in plants, if *TF* > 1, the Cd concentration in the shoot is higher than that in the root; otherwise, higher Cd concentrations are in the root rather than in the shoot. For showing the state of physiological symptoms, if *TF* > 1, the physiological traits will be induced more in the shoot; otherwise, they will be induced more in the root. Based on the steady-state of physiological symptoms with CK, one needs an approach to differentiate the changes in the physiological state by Cd treatment for illustration of the physiological regulations in rice plants under Cd stress. A tendency index (*TI*) was then derived with the ratio of the *TF* value for Cd-treated specimens to that for CK:

4 $$ TI = \frac{{ TF_{Cd - treated} }}{{TF_{CK} }} . $$

If *TI* > 1, compared with CK, Cd treatment will enhance physiological traits that are more pronounced toward the shoot; otherwise, it will enhance physiological traits that are more pronounced toward the root. The *TI* values indicating the differences in the physiological traits between Cd treatment (i.e., exposed to 50 μM Cd for 7 days) and CK could thus be used for evaluating the Cd toxicity and distributions in rice plants associated with physiological regulations under Cd stress.

### Statistical analyses

The observed data of root elongation, shoot extension, Cd concentrations in root and shoot, *TF* values, MDA and H_2_O_2_ concentrations, and SOD and APX activities for the seedlings of the rice cultivars were analyzed. Analysis of variance (ANOVA) was performed by using the GLM process of the SAS program (SAS Institute Inc. 1985) for testing the genotypic effects of Cd treatment (i.e. 50 µM Cd for 7 days) on plant growth, Cd-accumulation and -translocation, oxidative stress and damage, and antioxidant enzymes. By the F-test in one-way ANOVA, significance testing was carried out for the difference between the means, which were derived from the three replicates (*n *= 3) in the hydroponic experiment, of any two cultivars with the last significant difference (LSD) test at *p* < 0.05. Moreover, in order to infer the relative effects of Cd treatment compared with CK on the root elongation, shoot extension, Cd concentrations in root and shoot, MDA and H_2_O_2_ concentrations, and SOD and APX activities, Student’s t-test was carried out to make the comparisons between Cd treatment and CK for each cultivar. Also, the principle component analysis (PCA) was carried out by using the PRINCOMP process of the SAS program (SAS Institute Inc. 1985) to integrate the variations in Cd toxicity, absorption, and physiological regulation among the rice cultivars. The data of 12 variants for plant growth, Cd distribution and physiological traits responding to the Cd stress (i.e. *RRE*, *RSE*, Cd concentrations in root and shoot, and relative MDA, H_2_O_2_, SOD, and APX for root and shoot) were used in the PCA. The relative MDA, H_2_O_2_, SOD, and APX were obtained by using the ratio of the observation for Cd treatment to that in CK.

## Results

### Cadmium concentrations and translocation in rice plant

**Fig. 1:**
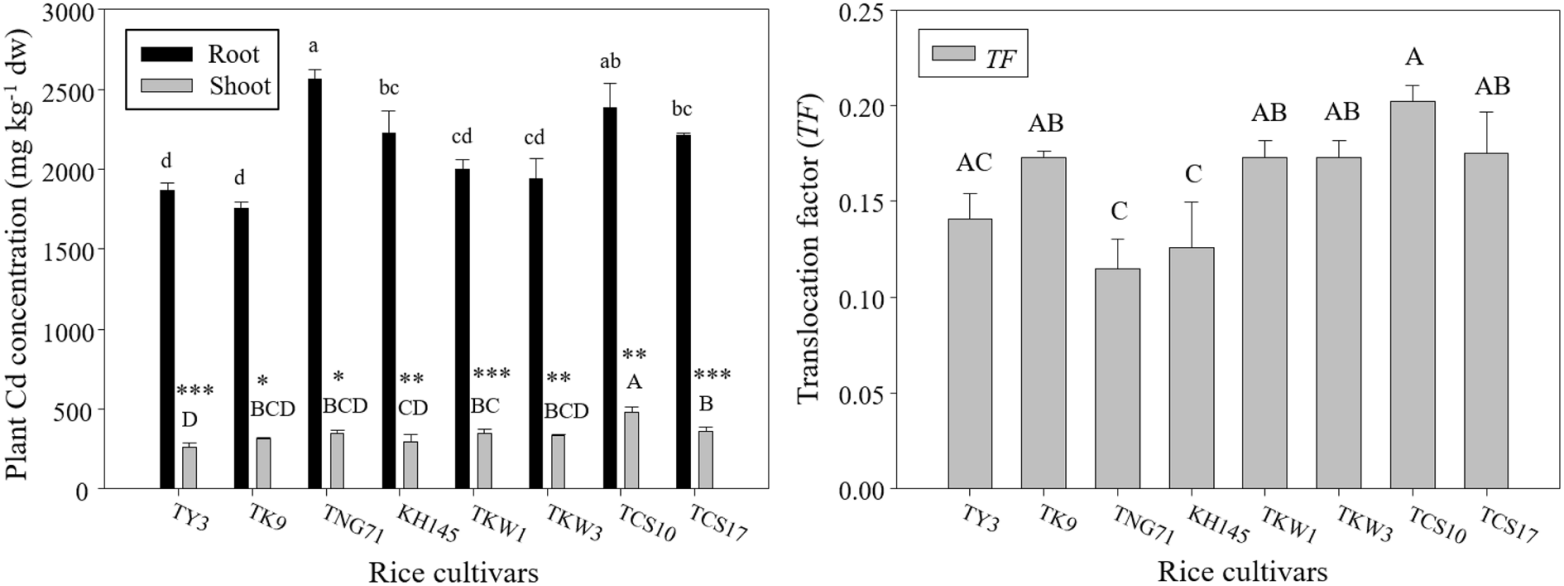
Concentrations of Cd in root and shoot and translocation factor (*TF*) of the seedlings after a 7-day exposure of 50 μM Cd (Cd treated) for the rice cultivars (i.e. TY3, TK9, TNG71, KH145, TKW1, TKW3, TCS10, and TCS17). Error bars represent the standard errors of the mean values (replicates *n* = 3). Same lowercase letters above the black bars and same capital letters above the gray bars indicate the mean values are not significantly different at *p *< 0.05 among the cultivars according to the last significant difference (LSD). *, **, and *** indicate significant differences at *p *< 0.05, *p *< 0.01, and *p *< 0.001 between root and shoot Cd concentrations by the t-test

Figure 1 shows the Cd concentrations in root and shoot for the used cultivars and the *TF* values were also obtained for illustrating Cd translocation in plants. According to the ANOVA, the genotypic effects of Cd treatment on plant Cd concentrations in root and shoot and on *TF* values were significant, and the multiple comparison by LSD testing was shown in Fig. 1. In CK, the Cd concentrations in the root and shoot for each cultivar were not detectable. With Cd treatment, Cd concentrations in the root were 1800–2600 mg kg^−1^ dw, and those in the shoot were lower than 500 mg kg^−1^ dw. The profiles of *TF* values for the rice seedlings after 7-day exposure of 50 μM Cd ranged from 0.1 to 0.2. There were significant deviations among the *TF* values for the rice cultivars. *TF* values of TNG71 and KH145 were relatively low, being less than even 0.15; by contrast, TCS10 showed the highest *TF* of ~ 0.2.

### Reductions in plant growth by Cd treatment

**Fig. 2:**
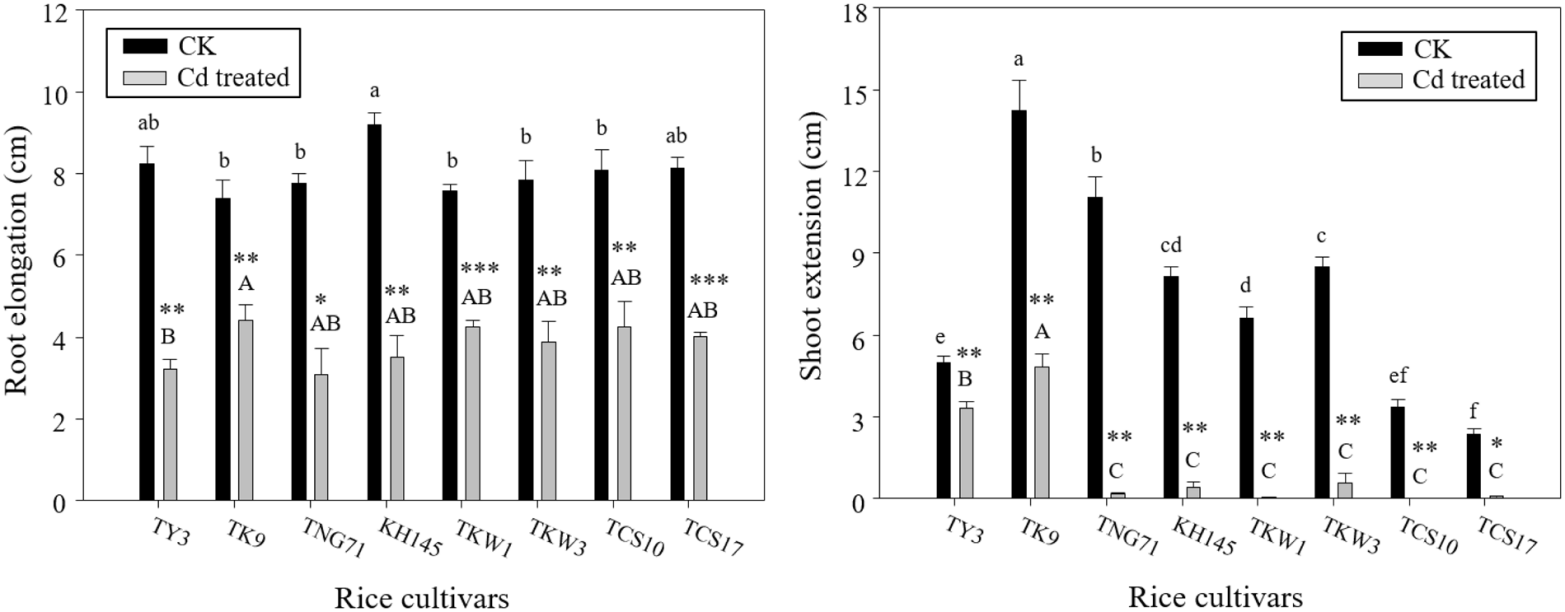
Root elongation and shoot extension of the seedlings after a 7-day exposure of 50 μM Cd (Cd treated) for the rice cultivars, TY3, TK9, TNG71, KH145, TKW1, TKW3, TCS10, and TCS17, and making comparison with a check (CK) for root elongation and shoot extension. Error bars represent the standard errors of the mean values (replicates *n* = 3). Same lowercase letters above the black bars and same capital letters above the gray bars indicate the mean values are not significantly different at *p *< 0.05 among the cultivars according to the last significant difference (LSD). *, **, and *** indicate significant differences at *p *< 0.05, *p *< 0.01, and *p *< 0.001 between Cd treated and CK by the t-test

Figure 2 shows the growth responses to Cd treatment with root elongation and shoot extension. The effects of Cd treatment on root elongation and shoot extension among the cultivars were significant in testing by the ANOVA. The root elongations for all cultivars were ~ 8 cm in CK and ~ 4 cm with Cd treatment. The Cd treatment resulted in significant reductions in the root growth for all of the cultivars. The reduction in root elongation by Cd treatment was less in TK9 and more in TY3. Moreover, according to the shoot extension profiles, there was a great variation in shoot growth among the cultivars. In CK, the shoot extensions of TK9, TNG71, KH145, TKW1, and TKW3 were higher than 6 cm, but those of TY3, TCS10, and TCS17 were not. Under Cd treatment, TNG71, KH145, TKW1, TKW3, TCS10, and TCS17 cultivars showed shoot extensions that were lower than 1 cm or were undetectable. That is, their shoot growths had high degrees of injury with Cd treatment. However, both TY3 and TK9 showed shoot extension over 3 cm under Cd treatment; that is, their shoot growths had lower degrees of injury.

### Oxidative statuses and antioxidant enzymes in rice plant

**Fig. 3:**
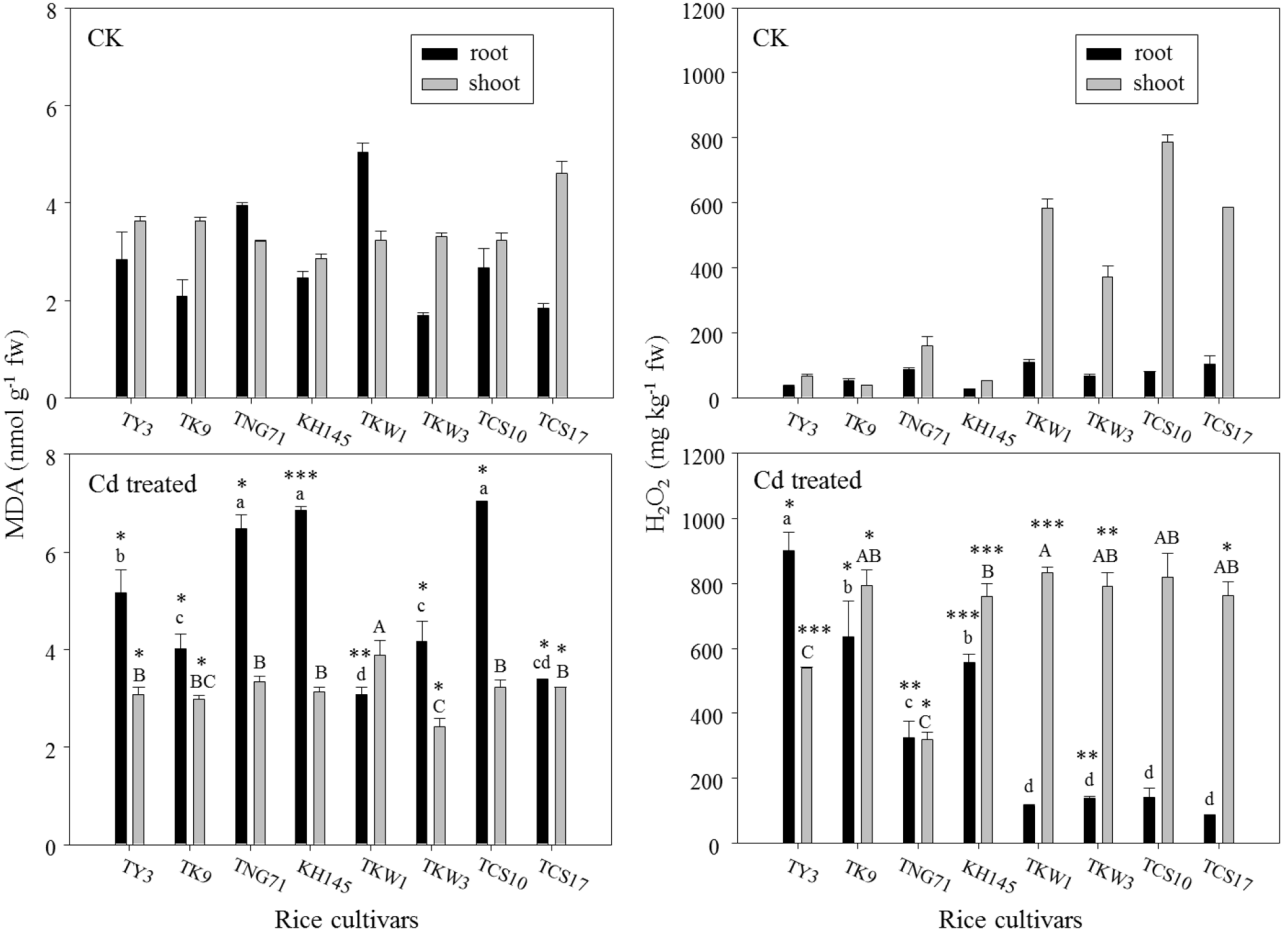
Concentrations of malondialdehyde (MDA) and H_2_O_2_ in root and shoot for the rice seedlings after a 7-day exposure of 50 μM Cd (Cd treated) and a check (CK) of the cultivars, TY3, TK9, TNG71, KH145, TKW1, TKW3, TCS10, and TCS17. Error bars represent the standard errors of the mean values (replicates *n* = 3). Same lowercase letters above the black bars or same capital letters above the gray bars indicate the mean values are not significantly different at *p *< 0.05 among the cultivars according to the last significant difference (LSD). *, **, and *** indicate significant differences at *p *< 0.05, *p *< 0.01, and *p *< 0.001 between Cd treated and CK by the t-test

Figure 3 shows the profiles of MDA and H_2_O_2_ concentrations in the root and shoot for the rice cultivars. The variations in MDA and H_2_O_2_ concentrations in the root and shoot among the cultivars were tested by using ANOVA and LSD test. Compared with CK, Cd treatment resulted in significant changes in the root MDA. The lipid peroxide levels in the root for all cultivars except TKW1 were significantly enhanced by Cd treatment. After Cd treatment, the root MDA concentrations of TNG71, KH145, and TCS10 were above 6 mmol g^−1^ fw and higher than the other cultivars’ root MDA concentrations. However, Cd treatment seemingly resulted in a reduction of shoot MDA for the cultivars. The shoot MDA concentrations of TY3, TK9, TKW3, and TCS17 were even lower than 4 mmol g^−1^ fw. That is, the lipid peroxide levels in the shoot for all cultivars did not increase with Cd treatment.

In CK, the H_2_O_2_ concentrations in the root for all cultivars were lower than 100 mg kg^−1^ fw. After Cd treatment, the root H_2_O_2_ concentrations of TY3, TK9, TNG71, and KH145 increased significantly and were 300–900 mg kg^−1^ fw; however, those for TKW1, TKW3, TCS10, and TCS17 were lower than 200 mg kg^−1^ fw. Further, the root H_2_O_2_ concentrations of TKW1, TCS10, and TCS17 were not enhanced significantly by Cd treatment. By contrast, with Cd treatment, the H_2_O_2_ concentrations in the shoot for the cultivars excepting TCS10 were in the range of 300–800 mg kg^−1^ fw and were increased significantly.

**Fig. 4:**
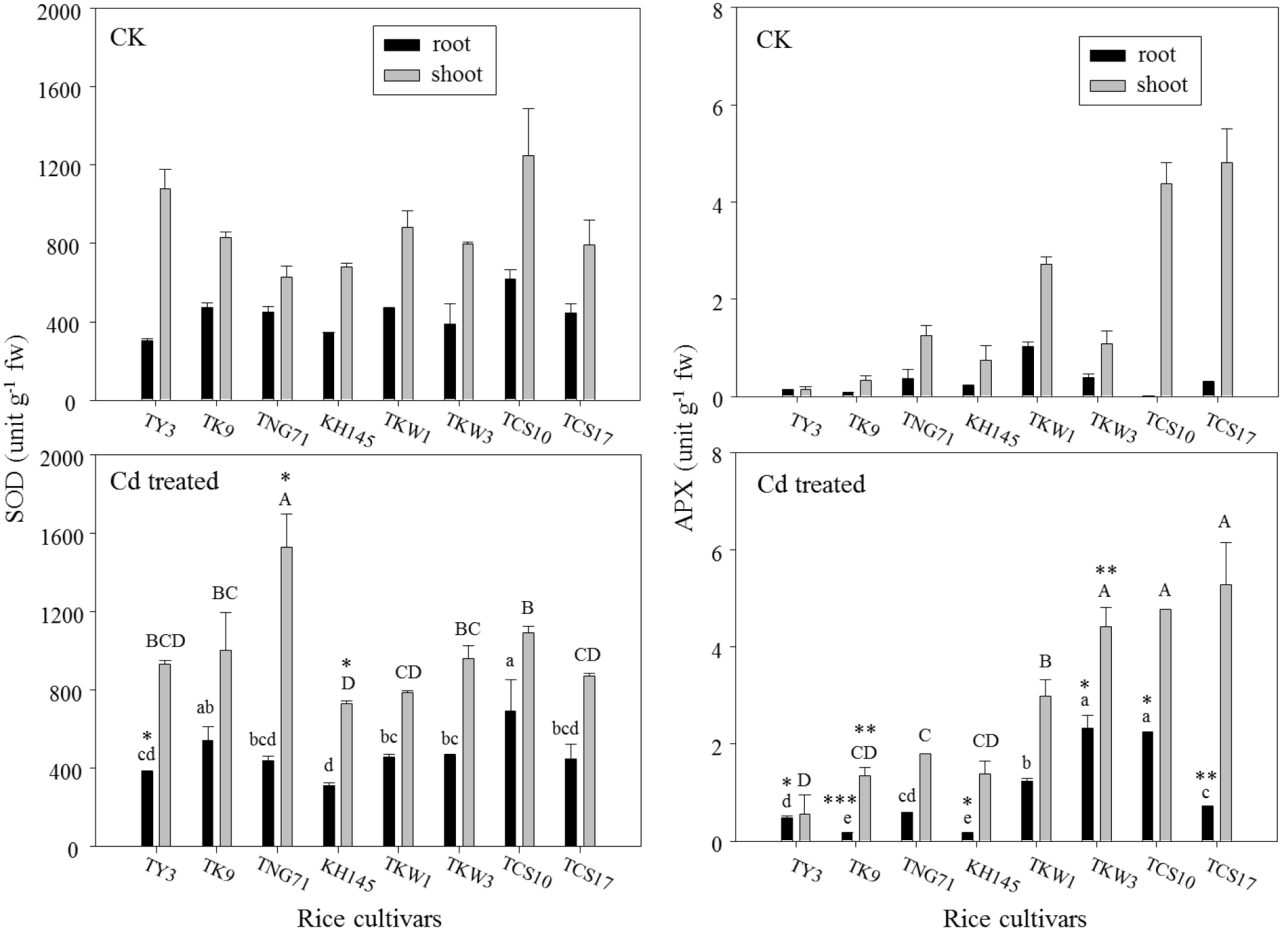
Activities of superoxide dismutase (SOD) and ascorbate peroxidase (APX) in root and shoot for the rice seedlings after a 7-day exposure of 50 μM Cd (Cd treated) and a check (CK) of the cultivars, TY3, TK9, TNG71, KH145, TKW1, TKW3, TCS10, and TCS17. Error bars represent the standard errors of the mean values (replicates *n* = 3). Same lowercase letters above the black bars and same capital letters above the gray bars indicate the mean values are not significantly different at *p *< 0.05 among the cultivars according to the last significant difference (LSD). *, **, and *** indicate significant differences at *p *< 0.05, *p *< 0.01, and *p *< 0.001 between Cd treated and CK by the t-test

Figure 4 shows the activities of both antioxidant enzymes, SOD and APX, in rice plants after Cd treatment and in CK. The variations in MDA and H_2_O_2_ concentrations in the root and shoot among the cultivars were tested by using ANOVA and LSD test. For all cultivars, the SOD and APX activities in the shoot were higher than those in the root. Significant increases in SOD activity with Cd treatment were found only in the root of TY3 and in the shoots of TNG71 and KH145. And significant increases in APX activity with Cd treatment were found in the roots for the cultivars except for TNG71 and TKW1; these increases were seen in the shoots of only TK9 and TKW3.

### PCA for plant growth, Cd accumulation and physiological traits

Table 1 shows the eigenvectors and eigenvalues for the first four principal components (PC1, PC2, PC3, and PC4) derived from the 12 variants: *RRE*, *RSE*, Cd concentrations in root and shoot (Cd/r and Cd/s), and relative MDA, H_2_O_2_, SOD, and APX in root and shoot (MDA/r, MDA/s, H_2_O_2_/r, H_2_O_2_/s, SOD/r, SOD/s, APX/r, and APX/s). According to the eigenvalues for PC1, PC2, PC3, and PC4, their cumulative proportion to the 12 variants approached to 0.85. By using PC1, PC2, PC3, and PC4, one can explain about 85% of the variations in plant growth, Cd accumulation and physiological traits among the rice cultivars.

**Table 1:** Eigenvectors and eigenvalues for the principal components (PC1, PC2, PC3, and PC4) derived from the variants: *RRE*, *RSE*, Cd concentrations in root and shoot (Cd/r and Cd/s), and relative MDA, H_2_O_2_, SOD, and APX in root and shoot (MDA/r, MDA/s, H_2_O_2_/r, H_2_O_2_/s, SOD/r, SOD/s, APX/r, and APX/s)

Eigenvectors	PC1	PC2	PC3	PC4
*RRE*	− 0.1731	0.5049	0.0771	− 0.2975
*RSE*	0.4088	0.0353	0.0810	0.0199
Cd/r	− 0.3804	− 0.1160	0.0248	0.2456
Cd/s	− 0.3399	0.0403	0.0255	0.1003
MDA/r	− 0.0154	− 0.0208	− 0.6365	0.4529
MDA/s	− 0.1973	− 0.2640	0.5915	− 0.0461
H_2_O_2_/r	0.3552	− 0.2538	0.2140	0.3310
H_2_O_2_/s	0.2074	0.1081	0.3465	0.3627
SOD/r	0.3359	0.3968	− 0.0014	− 0.1216
SOD/s	− 0.1542	− 0.2479	− 0.0688	− 0.4204
APX/r	− 0.2164	0.3808	0.2468	0.4207
APX/s	0.3884	0.2382	0.0025	− 0.1544
Eigenvalues	4.6986	2.5003	1.7246	1.3185
Proportion	0.3915	0.2084	0.1437	0.1009
Cumulative (%)	39.15	59.99	74.36	85.35

**Fig. 5:**
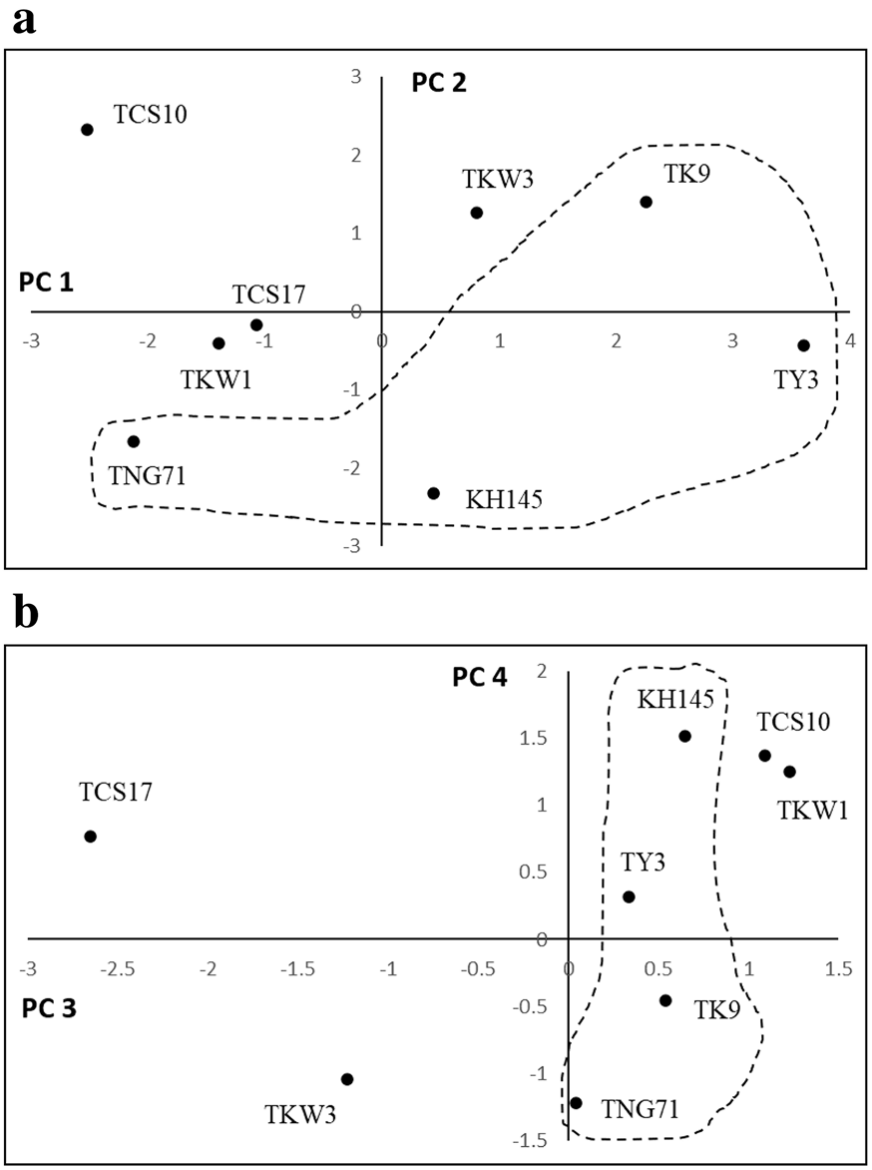
Biplots for the rice cultivars, TY3, TK9, TNG71, KH145, TKW1, TKW3, TCS10, and TCS17 on the coordinates **a** PC1 vs. PC2 and **b** PC3 vs. PC4, respectively. The first four principal components (i.e. PC1, PC2, PC3, and PC4), which were derived from the 12 variants for plant growth, Cd accumulation and physiological indices, explained 85.4% of the cultivars’ variance corresponding to the eigenvalues λ_1_ = 4.7, λ_2_ = 2.5, λ_3_ = 1.7, and λ_4_ = 1.3, respectively. The dash line delineates the group of TY3, TK9, TNG71, and KH145

In addition, the rice cultivars, TY3, TK9, TNG71, KH145, TKW1, TKW3, TCS10, and TCS17 were located on the both coordinates of PC1 vs. PC2 and PC3 vs. PC4, respectively (Fig. 5). In Fig. 5a, TK9, TNG71, TKW1, TKW3, and TCS17 are scattered around the diagonal line. However, TCS10 locates in the second quadrant, and TY3 and KH145 are in the fourth quadrant. In Fig. 5b, TY3, TK9, TNG71, KH145, TKW1, and TCS10 are sprinkled on the right side of PC4 axis; TCS17 locates in the second quadrant and TKW3 is in the third quadrant.

### Physiological regulations in rice plants under Cd stress

According to the abovementioned physiological traits (i.e., MDA, H_2_O_2_, SOD, and APX) in rice plants with Cd treatment (Figs. 3 and 4), the physiological patterns responding to Cd stress in the root were different from those in the shoot. The *TF* values of MDA, H_2_O_2_, SOD, and APX were used (Eq. (3)) to associate the responses to Cd stress in the root and shoot (Table 2). In CK, *TF* observations showed the steady-state conditions in which a delicate balance exists between the production of reactive oxygen species and the cellular antioxidative defense machinery (Gill and Tuteja 2010; Srivastava et al. 2015). Compared with the *TF* values in CK, those under Cd treatment indicated the disturbances in stationary oxidative statuses for rice seedlings. With Cd treatment, the *TF* values of both SOD and APX for all cultivars were much higher than 1.

**Table 2:** Genotypic variations in the translocation factor (*TF*) values for the selected physiological traits, malondialdehyde (MDA), H_2_O_2_, superoxide dismutase (SOD), and ascorbate peroxidase (APX), and in the tendency index (*TI*) values for the Cd treatment relative to a check (CK) among the rice cultivars

Rice cultivars	MDA			H_2_O_2_			SOD			APX		
	*TF*		*TI*	*TF*		*TI*	*TF*		*TI*	*TF*		*TI*
	CK	Cd		CK	Cd		CK	Cd		CK	Cd	
TY3	1.278	0.596	0.47	1.946	0.599	0.31	3.567	2.448	0.69	1.001	1.197	1.20
TK9	1.731	0.743	0.43	0.718	1.250	1.74	1.761	1.841	1.05	4.639	7.394	1.59
TNG71	0.816	0.515	0.63	1.863	0.981	0.53	1.402	3.488	2.49	3.414	3.049	0.89
KH145	1.156	0.456	0.39	1.975	1.364	0.69	1.994	2.357	1.18	3.297	8.690	2.64
TKW1	0.641	1.270	1.98	5.435	7.097	1.31	1.898	1.712	0.90	2.643	2.438	0.93
TKW3	1.962	0.581	0.30	5.700	5.667	0.99	2.029	2.055	0.99	2.811	1.894	0.67
TCS10	1.204	0.459	0.38	9.795	5.798	0.59	2.019	1.584	0.78	419.9	2.130	0.01
TCS17	2.493	0.950	0.38	5.619	8.536	1.52	1.773	1.946	1.10	15.98	7.283	0.46

In order to illustrate the physiological regulation in rice plants under Cd stress, which should be based on the steady-state of physiological symptoms with CK, the *TI* values, which indicate the tendency of changes in the physiological traits (i.e. MDA, H_2_O_2_, SOD, and APX) toward shoot by Cd treatment (Eq. (4)), for the rice cultivars were also shown in Table 2. All cultivars except TKW1 had the *TI* values of MDA being lower than 0.5. However, almost all cultivars had the *TI* values of H_2_O_2_ being higher than 0.5, except for TY3. The *TI* values of SOD for the cultivars except TY3 and TCS10 were close to or higher than 1. Also, most cultivars excluding TKW3, TCS10, and TCS17 had the *TI* values of APX were relatively near and higher than 1.

Moreover, Table 3 shows the correlation coefficients (*r*) between the plant growth factors, *RRE*, and *RSE* and the plant Cd concentrations in the root and shoot versus the *TI* values of SOD, APX, H_2_O_2_, and MDA. The *RRE* and *RSE* were not significantly correlated with the *TI* values of SOD, APX, H_2_O_2_, and MDA except for the relationship between *RRE* and *TI* of H_2_O_2_. The positive correlation between *RRE* and *TI* of H_2_O_2_ (*r* = 0.77) was significant.

**Table 3:** Correlation coefficients between the plant growth factors, relative root elongation (*RRE*) and relative shoot extension (*RSE*), and the plant Cd concentrations in root and shoot versus the tendency index (*TI*) values of physiological traits, superoxide dismutase (SOD), ascorbate peroxidase (APX), H_2_O_2_, and malondialdehyde (MDA)

*TI* values of physiological traits	Plant growth		Plant Cd	
	*RRE*	*RSE*	Root	Shoot
SOD	− 0.39	− 0.35	**0.65**	− 0.04
	(*p* > 0.30)^†^	(*p* > 0.30)^†^	**(*p*** **= 0.08)**	(*p* > 0.30)
APX	− 0.36	0.24	− 0.25	− **0.73**
	(*p* > 0.30)	(*p* > 0.30)	(*p* > 0.30)	**(*p*** **= 0.04)**
H_2_O_2_	**0.77**	− 0.20	− 0.44	0.01
	**(*p*** **= 0.02)**	(*p* > 0.30)	(*p* = 0.28)	(*p* > 0.30)
MDA	0.32	− 0.21	− 0.09	0.01
	(*p* > 0.30)	(*p* > 0.30)	(*p* > 0.30)	(*p* > 0.30)

The bold font highlights the significance at *p* < 0.1

^†^*p* values in parentheses indicate the significant levels

## Discussion

### The cultivar variations in Cd toxicity and distribution in rice plants

In Fig. 1, with Cd treatment, the Cd concentrations in the root would be higher than 2000 mg kg^−1^ dw, and those in the shoot were approaching to 500 mg kg^−1^ dw. The ranges of Cd concentrations in rice seedlings were also found in the recent studies by using (Chiao et al. 2019; Li et al. 2019). The *TF* values, that almost all were less than 0.2, indicated that most of the Cd uptake by rice plants was accumulated in the root rather than being transferred to the shoot. Previous studies have emphasized that Cd uptake in the root dominates Cd accumulation in the overall rice plant (Dong et al. 2007; Takahashi et al. 2011; Ye et al. 2012). Zhang et al. (2009) suggested that the detoxification of Cd in rice plants could be referred to a succession of Cd retention in root cell walls (Xiong et al. 2009; Liu et al. 2016), compartmentalization of Cd into vacuoles (Ernst et al. 2008; Zhang et al. 2013), and suppressed Cd transportation from the root to shoot. The root Cd concentrations of TNG71 and TCS10 were significantly higher than 2000 mg kg^−1^ dw, but those of TY3 and TK9 were significantly lower. This revealed that the capability of rice roots to retain Cd varies with the rice cultivars.

In Fig. 2, for all used cultivars, root elongation varied only moderately with Cd treatment; by contrast, shoot extensions varied much more. The genotypic variation in Cd toxicity to rice seedlings would be related to the Cd distribution in plants and physiological regulations under Cd stress (Hsu and Kao 2003; Zhang et al. 2009; Singh and Shah 2014). TKW1, TKW3, TCS10, and TCS17, whose *TF* values were relatively high (i.e., > 0.15), showed serious injury in shoot extension with Cd treatment; however, TY3 and TK9 also had relatively high *TF* values but did not show severe injury. Both TY3 and TK9 show higher shoot extension under Cd treatment but may use other physiological regulations. The two cultivars, TY3 and TK9, possessed a low absorption level resulting in better shoot extension compared with other cultivars; thus, they would be more tolerant to Cd stress. By contrast, TNG71 and KH145 with relatively low *TF* values (i.e., < 0.15) could limit Cd accumulation in shoot but reduce growth in shoot extension with high Cd absorption levels. And the indica rice, TCS10 and TCS17, possessed both higher absorption by root and higher translocation capacity resulting in higher Cd accumulation in the shoot. The above findings suggested that the genetic diversity of Cd tolerance and distribution in plant would be very abundant.

Nevertheless, root-to-shoot Cd translocation is the major process determining grain Cd accumulation (Uraguchi and Fujiwara 2012). Wang et al. (2015) emphasized that stem transportation may play an important role in grain Cd accumulation, which was combined with the expression level of the OsPCR1 gene. Lower Cd translocation from the root to the shoot may result in lower grain Cd accumulation. Therefore, *TF* values could be used as an index in a selection program for initially screening cultivars whose edible parts (e.g., grain or brown rice) potentially have low Cd contents (Zhang et al. 2014; Song et al. 2015; Liu et al. 2016). According to the *TF* values of used cultivars in Fig. 1, TNG71 and KH145 show the lower potential of grain Cd accumulation although they are not tolerant of Cd stress. Nevertheless, TK9 not only tolerate Cd treatment such as 50 μM but also show the superior potential of grain Cd accumulation.

### Characterization of the oxidative statuses in rice plants under Cd stress

In previous studies (He et al. 2014; Wang et al. 2015), the MDA concentrations in the rice shoot increased significantly with the treatment of high Cd levels (i.e., 100–500 μM). Xie et al. (2015) reported that the MDA observed in the shoot of rice seedlings treated with low Cd concentrations of ~ 40 μM for 15 days increased by up to two times compared with that in the control. In this study, rice seedlings were treated with 50 μM Cd for 7 days; however, they did not show a significant enhancement of lipid peroxides in the shoot, probably because the roots accumulated most of the Cd absorbed by rice seedlings to prevent translocation into the shoot. Thus, the Cd exposure level and duration did not result in high lipid peroxides in the shoot. Hsu and Kao (2007) suggested that the differences in H_2_O_2_ accumulation in leaves between different rice cultivars were related to the genotypic variation in Cd tolerance. In Fig. 3, the Cd treatment produced the greatest enhancements in shoot H_2_O_2_ concentration in TY3, TK9, and KH145, although their shoot H_2_O_2_ concentrations in CK were much lower, even being under 100 mg kg^−1^ fw. In contrast, the shoot H_2_O_2_ concentrations of TKW1, TKW3, TCS10, and TCS17 in CK were relatively high in a range from 400 to 800 mg kg^−1^ fw. Their seedlings in CK may be under some oxidative stress; this was consistent with their shoot extensions in CK being relatively low (Fig. 2). Nevertheless, with the Cd treatment, TKW1, TKW3, TCS10, and TCS17 showed moderately pronounced increases in shoot H_2_O_2_ concentrations. A few studies showed that H_2_O_2_ accumulation and MDA content were positively correlated in rice plants with Cd treatment (Singh and Shah 2014; He et al. 2014; Wang et al. 2015). However, this was not in agreement with the results of the present study. It may be possible that significant increases in H_2_O_2_ accumulation in the shoot with Cd exposure have been found for the cultivars, but have not been directly related to their MDA measurements. That is, the Cd exposure (i.e. 50 μM Cd for 7 days) was high enough to result in significant increases of oxidative stress rather than in significant accumulation of lipid peroxides in the shoot.

According to the above discussion, the oxidative statuses of rice plants with Cd treatment are associated with the configuration of H_2_O_2_ distributed in the shoot and root. Based on the variations in H_2_O_2_ concentrations in the shoot and root among the rice cultivars, they could be classified into the two groups. One includes TY3, TK9, TNG71, and KH145, whose H_2_O_2_ concentrations in both the root and the shoot increased dramatically with Cd treatment; the other includes TKW1, TKW3, TCS10, and TCS17, whose H_2_O_2_ concentrations in the shoot are more than those in the root with Cd treatment (Fig. 3).

### The antioxidative enzymes in rice plants under Cd stress

In Fig. 4, the enhancements of APX activity with Cd treatment were more pronounced in the root than in the shoot. All cultivars could be divided into two groups according to the profile APX activities as well as based on the H_2_O_2_ concentrations in the shoot and root (Fig. 3). One group includes TY3, TK9, TNG71, and KH145, whose APX activities in the root were less than 1 unit g^−1^ fw and those in the shoot were less than 2 unit g^−1^ fw. The other group includes TKW1, TKW3, TCS10, and TCS17, whose APX activities in the root were higher than 1 unit g^−1^ and those in the shoot were higher than 4 unit g^−1^ fw. After Cd treatment, the APX activities in both the root and the shoot for TY3, TK9, TNG71, and KH145 were much lower than those for TKW1, TKW3, TCS10, and TCS17 (Fig. 4). Also, the SOD activities of the group including TY3, TK9, TNG71, and KH145 were enhanced more significantly by Cd treatment than those for the other one including TKW1, TKW3, TCS10, and TCS17.

It is well known that SOD catalyzes the conversion of superoxide anion to less toxic H_2_O_2_ and shows promise as the first line of defense against oxidative stress (Shah et al. 2001). The major sources of H_2_O_2_ in cells are mitochondria and chloroplasts, in which peroxisomes and glyoxysomes contain SOD that is responsible for H_2_O_2_ production (Jiménez et al. 1997; Dixit et al. 2001). APX is the most important enzyme in chloroplast for scavenging H_2_O_2_ by ascorbate converting into H_2_O and O_2_ (Sidhu et al. 2016). The APX activities in the root and leaf could be stimulated by Cd treatment to remove H_2_O_2_ (Dixit et al. 2001), and they would be involved in some additive function in the metal tolerance mechanism in plants. For the group including TY3, TK9, TNG71, and KH145, H_2_O_2_ concentrations in the root and shoot increased greatly with Cd treatment owing to relatively low increases in APX activity. For the other one including TKW1, TKW3, TCS10, and TCS17, Cd treatment resulted in relatively high increases in APX activities in the root. Thus, their H_2_O_2_ concentrations with Cd treatment in the root did not increase dramatically as much as those seen for TY3, TK9, TNG71, and KH145. Based on the catalyzation mechanism of SOD in the dismutation of superoxide into H_2_O_2_ (Cruz de Carvalho 2008), the more the enhancement of SOD activity, the more is the increase in H_2_O_2_ concentration. Thus, H_2_O_2_ concentrations in the shoot being higher than those in the root for almost all rice cultivars by Cd treatment (Fig. 3) would be due to the SOD activities in the shoot being higher than those in the root for all the cultivars by Cd treatment (Fig. 4).

### Inductive illustration for the rice genotypic variations in responding to Cd stress

In Table 1, PC1 was dominated by *RSE*, H_2_O_2_/r, SOD/r and APX/s in the positive, but by Cd/r and Cd/s in the negative; PC2 was dominated positively by *RRE*, SOD/r and APX/r. The PC1 was highly related to *RES* (loading = 0.4088) and PC2 would be mainly governed by *RRE* (loading = 0.5049) and the cumulative proportion for PC1 and PC2 was approaching to 60%. The biplot PC1 vs. PC2 could be used to illustrate more details about the genotypic variation in plant growth under Cd stress. In Fig. 5a, TY3, TK9, TNG71, and KH145 spread on the bottom right side of the diagonal line, and TKW1, TKW3, TCS10, and TCS17 were on the other side. TY3, TK9, TNG71, and KH145 were grouped together. This revealed that for the seedlings of the group TY3, TK9, TNG71, and KH145, the Cd stress resulted in more reductions in shoot extension than in root elongation; by contrast for the group TKW1, TKW3, TCS10, and TCS17, due to Cd stress the reduction of root elongation would be much more than that of shoot extension (Fig. 2). The delineation was consistent with the classification of the cultivars into the two groups based on the genotypic variations in H_2_O_2_ and APX in shoot and root (Figs. 3 and 4). In addition, the PC3 was dominated by MDA/s in the positive, however by MDA/r in the inverse direction; PC4 was dominated positively by Cd/r, MDA/r, H_2_O_2_/r, H_2_O_2_/s, and APX/r, but negatively by RRE and SOD/s. However, the sum of the proportions for PC3 and PC4 was about 25%. In Fig. 5b, the score values of PC3 for TY3, TK9, TNG71, and KH145 were closely ranged from 0 to 1; they were clustered near PC4 axis. This suggested that the changes of MDA in root and shoot by Cd stress for TY3, TK9, TNG71, and KH145 were similar; and this would be in support of grouping them together.

### Inferential understandings for the physiological regulation in rice plants

The results in Table 2 showed that the antioxidative machinery was pronounced more in the shoot than in the root; this agreed with a previous study by He et al. (2014). The fact that with Cd treatment the *TF* values of H_2_O_2_ concentration for all cultivars except TY3 and TNG71were higher than 1 revealed that H_2_O_2_ would be present more in the shoot than in the root. However, the *TF* values of MDA were less than 1 for all cultivars except TKW1. That is, MDA preferentially accumulated in the root with Cd treatment. Nevertheless, the conflict between MDA and H_2_O_2_ distributed in the root and shoot arises owing to the relatively high SOD and APX activities in the shoot (Fig. 4). The high APX activity in the shoot could scavenge H_2_O_2_ to reduce lipid peroxidation in the shoot (He et al. 2014), even though there was higher H_2_O_2_ accumulation in the shoot owing to the high SOD activity to transform more superoxide anion into H_2_O_2_. In addition, according to the assessment of the *TI* values for the cultivars, the rice plants under Cd stress would preferentially enhance MDA toward the root and regulate H_2_O_2_ accumulation toward the shoot. The Cd treatment could promote most cultivars to preferentially regulate SOD and APX toward the shoot.

The results in Table 3 revealed that under Cd stress, the rice cultivars regulated H_2_O_2_ accumulation toward shoot and their root could be with gradually keeping growth. The cultivars such as TK9 and TKW1 had relatively high *TI* values of H_2_O_2_ (i.e. 1.74 and 1.31), and their root elongations were consistent with their *TI* values of H_2_O_2_ to be higher than other cultivars’ (Fig. 2). In addition, the root Cd content was significantly positively correlated with the *TI* of SOD (*r* = 0.65); however, the shoot Cd content was significantly negatively correlated with the *TI* of APX (*r* = − 0.73). Recall Fig. 1 to find that TNG71.s Cd concentration in root was the highest and KH145’s Cd concentration in the shoot was approaching to the lowest. Their *TF* values of Cd concentration were the lowest. Also, we found that TNG71 had the highest *TI* value of SOD (i.e., 2.49); KH145 had the highest *TI* value of APX (i.e., 2.64). These findings indicated that Cd preferentially accumulated in the root would be involved in the regulation tendency of SOD toward the shoot; the regulation tendency of APX toward the shoot would be related to less translocation of Cd into the shoot.

## Conclusions

All used cultivars (i.e., TY3, TK9, TNG71, KH145, TKW1, TKW3, TCS10, and TCS17) showed only a moderate variation in root elongation with Cd treatment. Their shoot extensions varied much more. Thus, the reduction of shoot growth owing to Cd stress would be a sensitive index to delineate the Cd tolerances for different rice cultivars. The cultivars preferentially accumulated Cd in the root rather than shoot, and then had a significant increase of MDA and a reduction in root growth. Nevertheless, H_2_O_2_ accumulation would be regulated toward the shoot to retard shoot growth suddenly, then the root could keep a gradual growth. Besides, H_2_O_2_ accumulation toward the shoot may be due to SOD is responsible for H_2_O_2_ production. According to the PCA for assessing in detail about the genotypic variations in plant growth, Cd accumulation and physiological traits, the rice cultivars were reliably delineated into the two groups. One includes TY3, TK9, TNG71, and KH145; the other includes TKW1, TKW3, TCS10, and TCS17. The rice cultivars, which preferentially accumulate Cd in the root, would have the regulation tendency of SOD toward the shoot. Moreover, the regulation tendency of APX toward the shoot would reduce Cd translocation into the shoot.

## Data Availability

The data and materials used and analyzed in the current study can be provided by the corresponding author for scientific, non‑profit purposes.
